# Supplementary material for: Collagenase-Responsive Hydrogel Loaded with GSK2606414 Nanoparticles for Periodontitis Treatment through Inhibiting Inflammation-Induced Expression of PERK of Periodontal Ligament Stem Cells
Source: Pharmaceutics. 2023 Oct 20;15(10):2503. doi: 10.3390/pharmaceutics15102503 (PMC10609791; doi:10.3390/pharmaceutics15102503)
Supplement: Supplementary file 1 [file pharmaceutics-15-02503-s001.zip › pharmaceutics-2570480-supplementary.pdf]

Supporting Information

Collagenase-Responsive Hydrogel Loaded with GSK2606414 Nanoparticles for Periodontitis Treatment through Inhibiting Inflammation-Induced Expression of PERK of Periodontal Ligament Stem Cells

1. Synthetic route and characterization of PDLLA-PEG-PDLLA

The synthetic route of PDLLA-PEG-PDLLA is shown in **Scheme S1**. The NMR hydrogen spectrum data of PDLLA-PEG-PDLLA are shown in **Figure S2**.

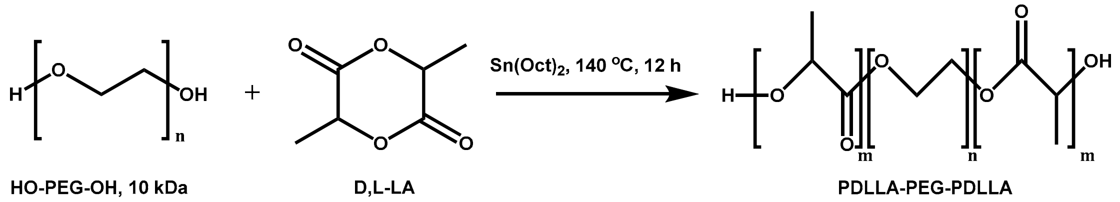

Scheme S1. Synthetic route of poly (D,L-lactide)-poly (ethylene glycol)-poly (D,L-lactide) (PDLLA-PEG-PDLLA).

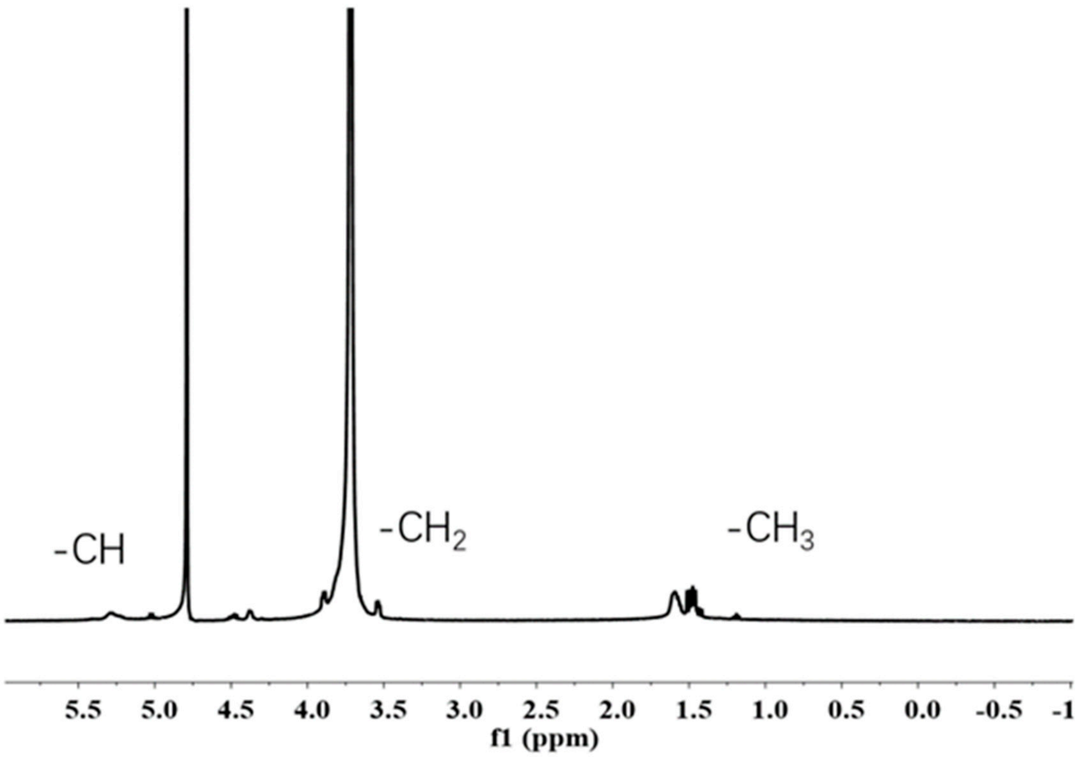

Figure S1 ^{1}H NMR spectra of PDLLA-PEG-PDLLA using CDCl3 as solvent and the positions of the characteristic peaks of each group are labeled.

2. Zeta Potential

The potential data of FreeGSK and NanoGSK are shown in **Table S1**.

Table S1 Potential values of FreeGSK and NanoGSK.

	Zeta Potential (mV)
**FreeGSK**	-3.92
**NanoGSK**	-8.83

3. GSK2606414 standard curve

By scanning GSK2606414 solution at 200-800 nm, its maximum absorption wavelength is 300 nm. After scanning solution with different concentration gradient, standard curve is obtained by linear fitting according to the relationship between Abs and concentration (as shown in **Figure S2**):

$$\mathrm{Abs} = 0.04548 \times \mathrm{c} + 0.002539 \quad \mathrm{R}^2 = 1.0000$$

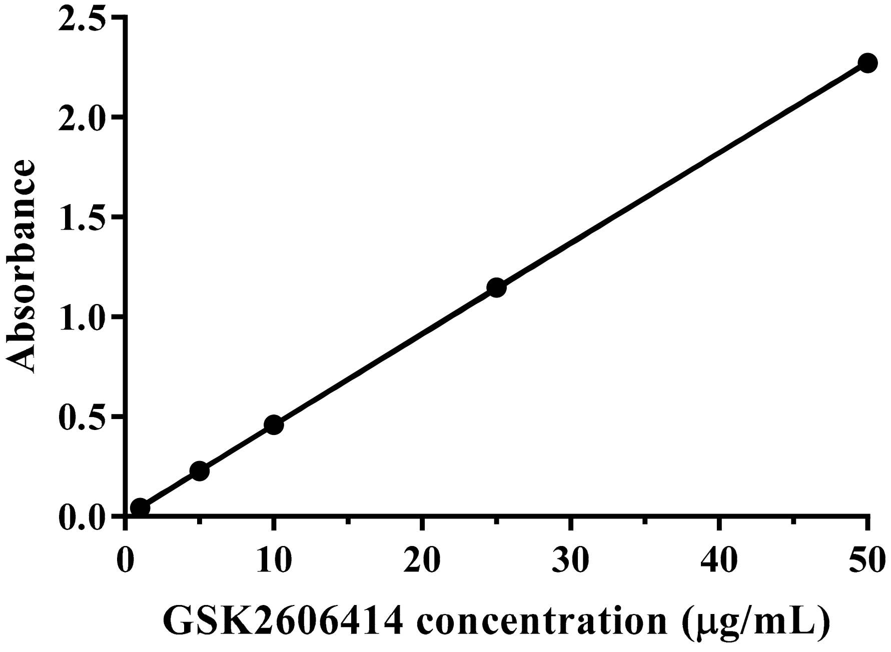

Figure S2 Standard curve of GSK2606414 with DMSO as solvent and absorbance measurement by UV-vis.

4. Synthetic route and characterization of HA-AC

The synthetic route of HA-AC is shown in **Scheme S2**. The NMR hydrogen spectrum data of HA-AC are shown in **Figure S3**.

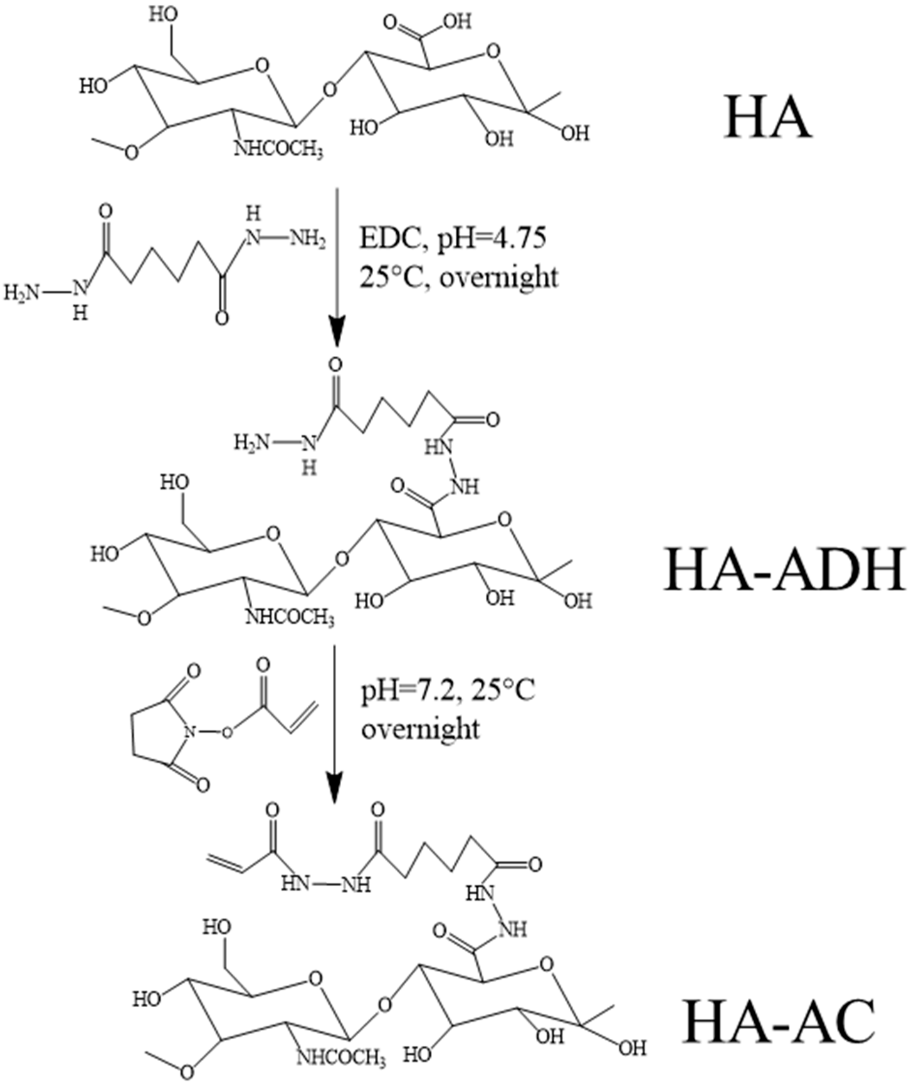

Scheme S2 Synthesis lines of hydrogel precursor materials HA-ADH and HA-AC.

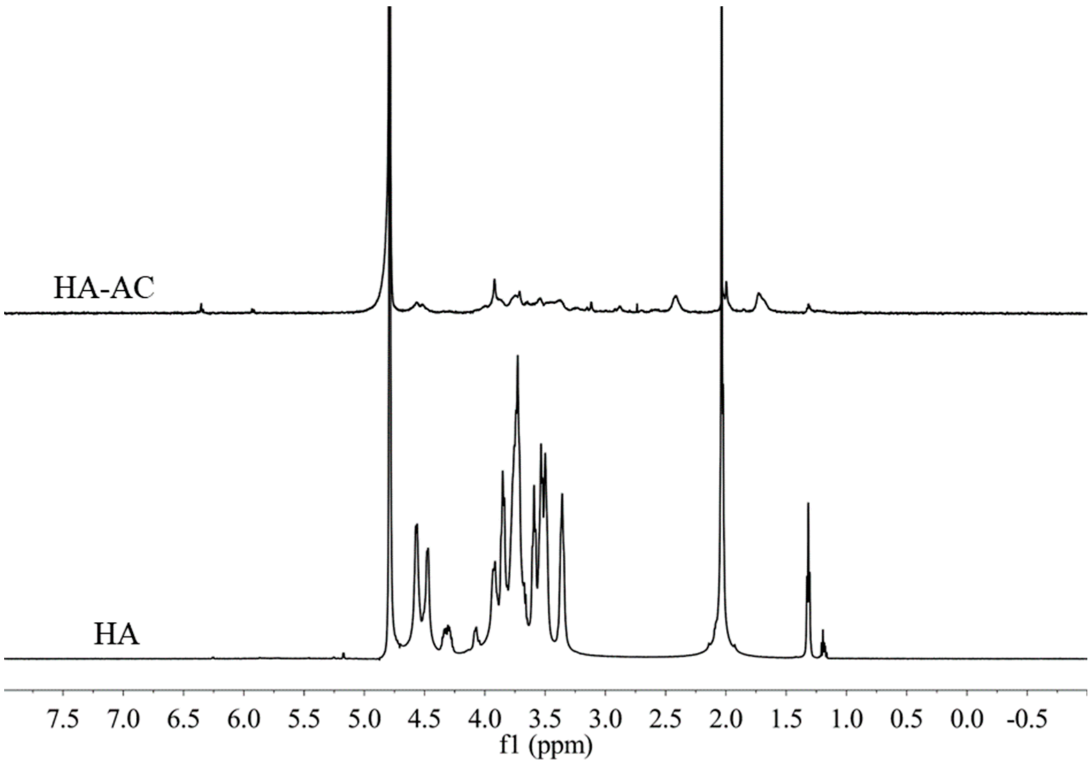

Figure S3 ^{1}H NMR spectra of HA and HA-AC with D_2O as solvent.

5. Cell culture photos

The photographs of the cell culture under different culture conditions are shown in **Figure S4**.

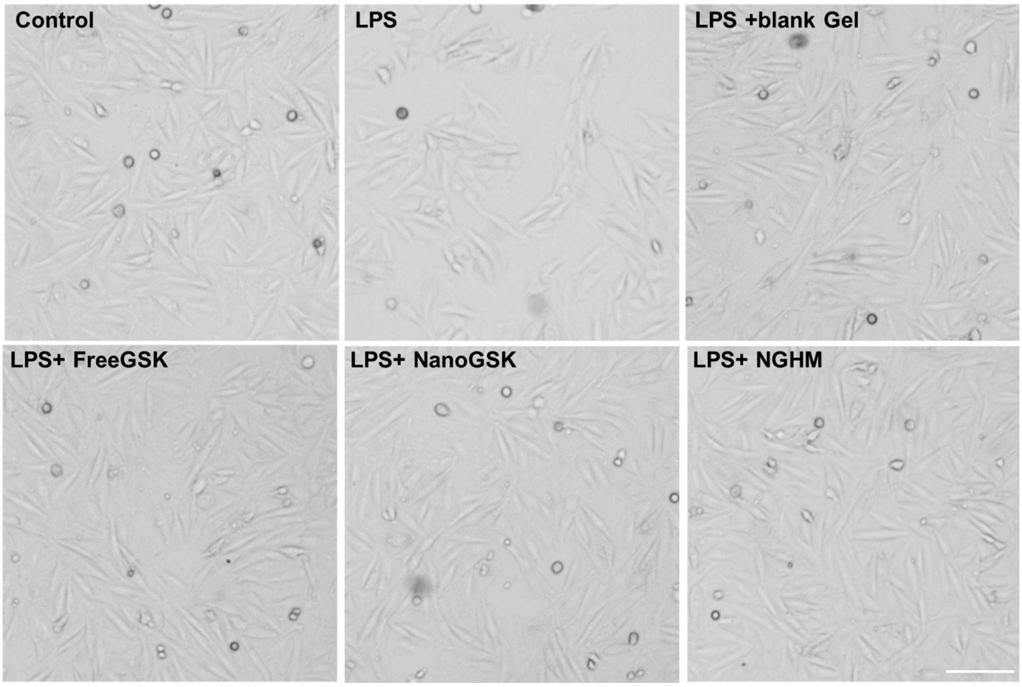

Figure S4 Cell status of PDLSCs after incubation under different conditions (Control, LPS, LPS+blank Gel, LPS+Free GSK, LPS+NanoGSK, LPS+NGHM, scale bar = 50 μm).
